# Persistent Differences in Mortality Patterns across Industrialized Countries

**DOI:** 10.1371/journal.pone.0106176

**Published:** 2014-09-02

**Authors:** Hippolyte d'Albis, Loesse Jacques Esso, Héctor Pifarré i Arolas

**Affiliations:** 1 Paris School of Economics - University Paris 1, Paris, France; 2 ENSEA, Abidjan, Côte d'Ivoire; 3 Toulouse School of Economics, University Toulouse 1, Toulouse, France; University of Arkansas, United States of America

## Abstract

The epidemiological transition has provided the theoretical background for the expectation of convergence in mortality patterns. We formally test and reject the convergence hypothesis for a sample of industrialized countries in the period from 1960 to 2008. After a period of convergence in the decade of 1960 there followed a sustained process of divergence with a pronounced increase at the end of the 1980's, explained by trends within former Socialist countries (Eastern countries). While Eastern countries experienced abrupt divergence after the dissolution of the Soviet Union, differences within Western countries remained broadly constant for the whole period. Western countries transitioned from a strong correlation between life expectancy and variance in 1960 to no association between both moments in 2008 while Eastern countries experienced the opposite evolution. Taken together, our results suggest that convergence can be better understood when accounting for shared structural similarities amongst groups of countries rather than through global convergence.

## Introduction

Whether there is global convergence in well-being across countries remains in the realm of scientific debate. The hypothesis of convergence has been studied for a variety of dimensions of well-being, with mixed results. While economic convergence is not a general reality [1], recent research has emphasized the existence of global demographic convergence [2] — in life-expectancy and fertility — and its importance for reductions in the inequality in living conditions [3]. We investigate the evolution of mortality patterns for a large group of industrialized countries through an analysis of their ages-at-death distributions. The ages-at-death distribution is given by the number of deaths at every given age in the period life table.

Our research advances the current understanding of mortality convergence by, first, formally testing the implications of the theory on the epidemiological transition, then second, uncovering trends in mortality patterns beyond the evolution of the mean, i.e. life expectancy, and variance of the considered distribution. Our chosen indicator of divergence, the Kullback-Leibler divergence (KLD), provides a comprehensive measure of the overall differences between distributions.

Studies of convergence classify differences between countries using two broad categories: first there is consideration for the unequal positions of countries in the stages of their development; second are structural differences, dissimilarities that persist even should countries become equally developed [1]. Thus far, expectations for convergence in mortality have been based on a catching-up process between countries in different stages of development. In particular, the hypothesis of convergence in mortality patterns has been a natural corollary to the theory of epidemiological transition [4], whereby countries lagging in their transition paths experience relatively faster gains in life expectancy and catch up with countries in the later stages [5], [6]. This is a consequence of the first stage in development whereby reductions of death rates arise from reductions in infectious diseases, a phenomenon with greater relative impact among infants. However, once the the reduction of infant mortality and relatively easily preventable deaths at younger adult ages have been realized, further gains in life expectancy are due to gains at older ages, which are increasingly costly and occur at much slower rates.

In this work, we test and reject the convergence hypothesis for industrialized countries in the period 1960–2008. However, we acknowledge that the lack of convergence for our whole sample does not necessarily imply that there are not subgroups of countries converging. In fact, the concept of convergence among subgroups or clubs of countries has already received some attention in the mortality convergence literature [7]. The basic theoretical difference is that proponents of the hypothesis of convergence club postulate that there might exist not one but several long term ages-at-death distributions. To address this possibility, we divide our sample in two groups, Eastern and Western countries, based on former pertinence to a common political history. We find that the trajectories of the two groups of countries are remarkably different. These results point out that the overall divergence trend is partly driven by trends in the differences between the two subgroups.

Our findings on the lack of convergence are coherent with recent findings that highlight the relatively high variability of mortality at young adult ages across countries and its contribution to international differences in mortality patterns [8], [9]. While the reduction of mortality at earlier life stages described by the theory of the epidemiological transition has contributed to convergence, differences in young adult mortality have acted as a countervailing force. This work contributes to the discussion on international mortality convergence by placing a special emphasis in both studying changes in the overall ages-at-death dstribution and recognizing the relevance of convergence clubs.

## Materials and Methods

Our object of study, mortality patterns, is extracted from the period life-tables available in the Human Mortality Database [10]. Following the work by Edwards and Tuljapurkar [8], we evaluate the dissimilarities between the ages-at-death distributions of all our countries with the Kullback-Leibler divergence (KLD), a measure of the overall dissimilarities between distributions. An advantage of using this index of dissimilarity is that we are no longer restricted to the study of the mortality distribution through its first two moments — life-expectancy and variance — which is particularly relevant given the non-normality of ages-at-death distributions. We describe any group of countries converging in mortality if the dissimilarities across their ages-at-death distributions diminish. Since the KLD is a measure of pairwise differences, we compute each sum of KLDs between individual countries and the period's average distribution and study its evolution. As we are only concerned with the trend (and not levels), this is formally equivalent to computing the pairwise sum of differences across countries.

The divergence of the age-at-death distribution for country 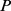 from that of country 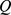 is given by the expression:
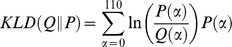
where 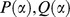 are the probability masses in each age group 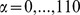. The higher the value of 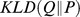, the larger the differences between two distributions. In our exercise, the average age-at-death distribution is computed as the unweighted average of ages-at-death: for every age group 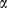, the arithmetic mean was computed. For a given sample of size 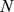, where 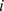 denotes a country, our measure of dispersion can be written as:
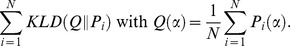



Instead of focusing on the particular value of the KLD, in our exercise we set 1960 as the base year.

Motivated by concerns of structural similarities and dissimilarities, we turn to the well-known literature on the effect of political and economic transitions of former Soviet countries, a process which has exerted a major influence in the form of a mortality shock for said countries [11], [12]. In order to perform an exploration of the importance of convergence clubs, we split the sample in two large groups: Western and Eastern countries (Table 1). Countries within Eastern Europe that experienced communism belong to our Eastern group of countries. In the next section, we first present a graphical analysis of the mean and variance for both samples then comment on the evolution of the KLD in the period 1960–2008.

**Table 1:** Country List.

Western countries		Eastern countries
Australia	Luxemburg	Belarus
Austria	Netherlands	Bulgary
Belgium	New Zeland	Czech Republic
Canada	Norway	East Germany
Denmark	Portugal	Estonia
Finland	Sweden	Hungary
France	United Kingdom	Latvia
Iceland	US	Poland
Ireland	West Germany	Russia
Italy		Slovakia
Japan		Ukraine

## Results

### Mean-variance analysis

**Figure 1:**
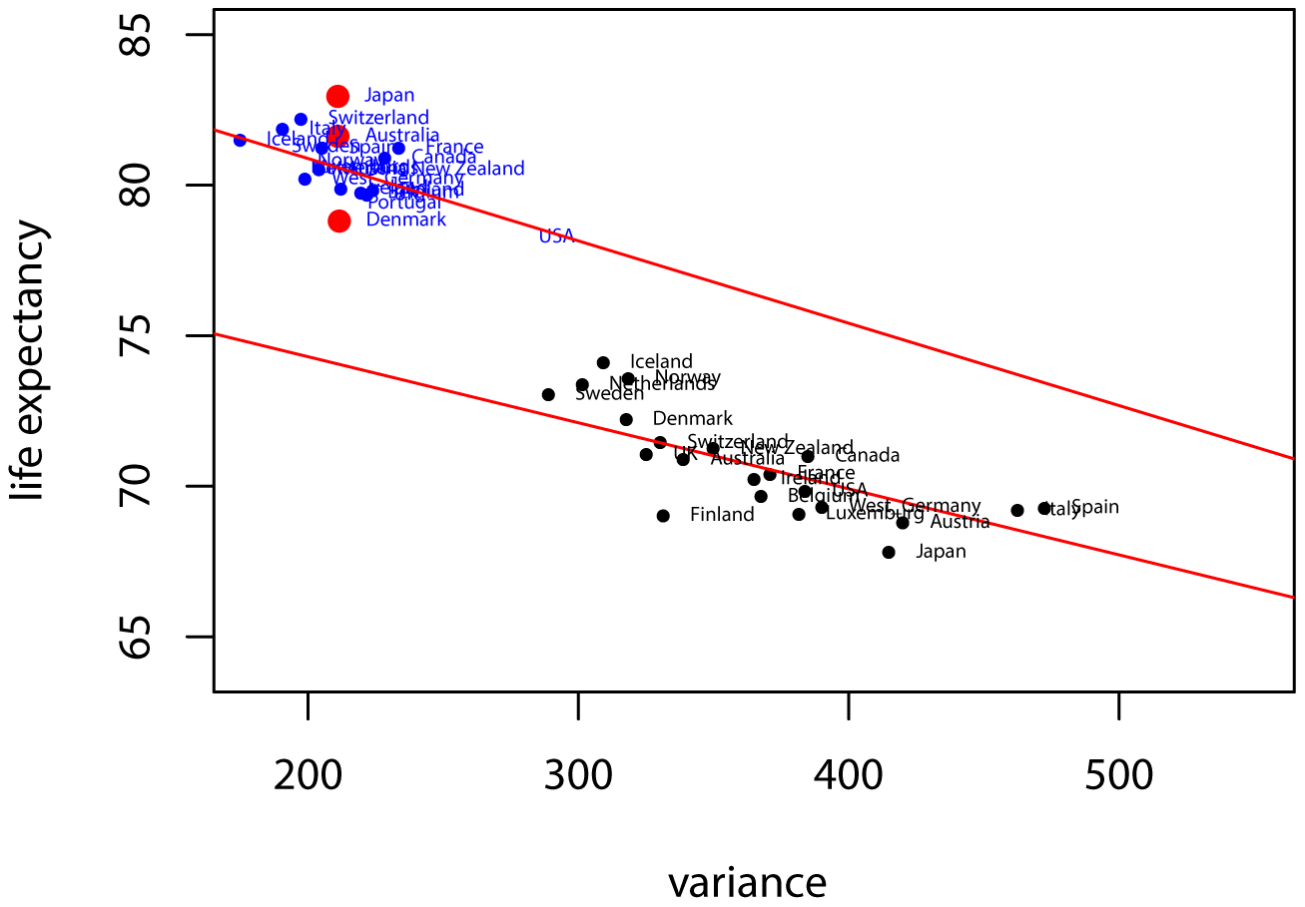
Variance and life expectancy profiles of Western countries. In blue, the variance and life expectancy profile of Western countries in the year 2008 and in black for the year 1960. In red, Japan, Australia and Denmark.

**Figure 2:**
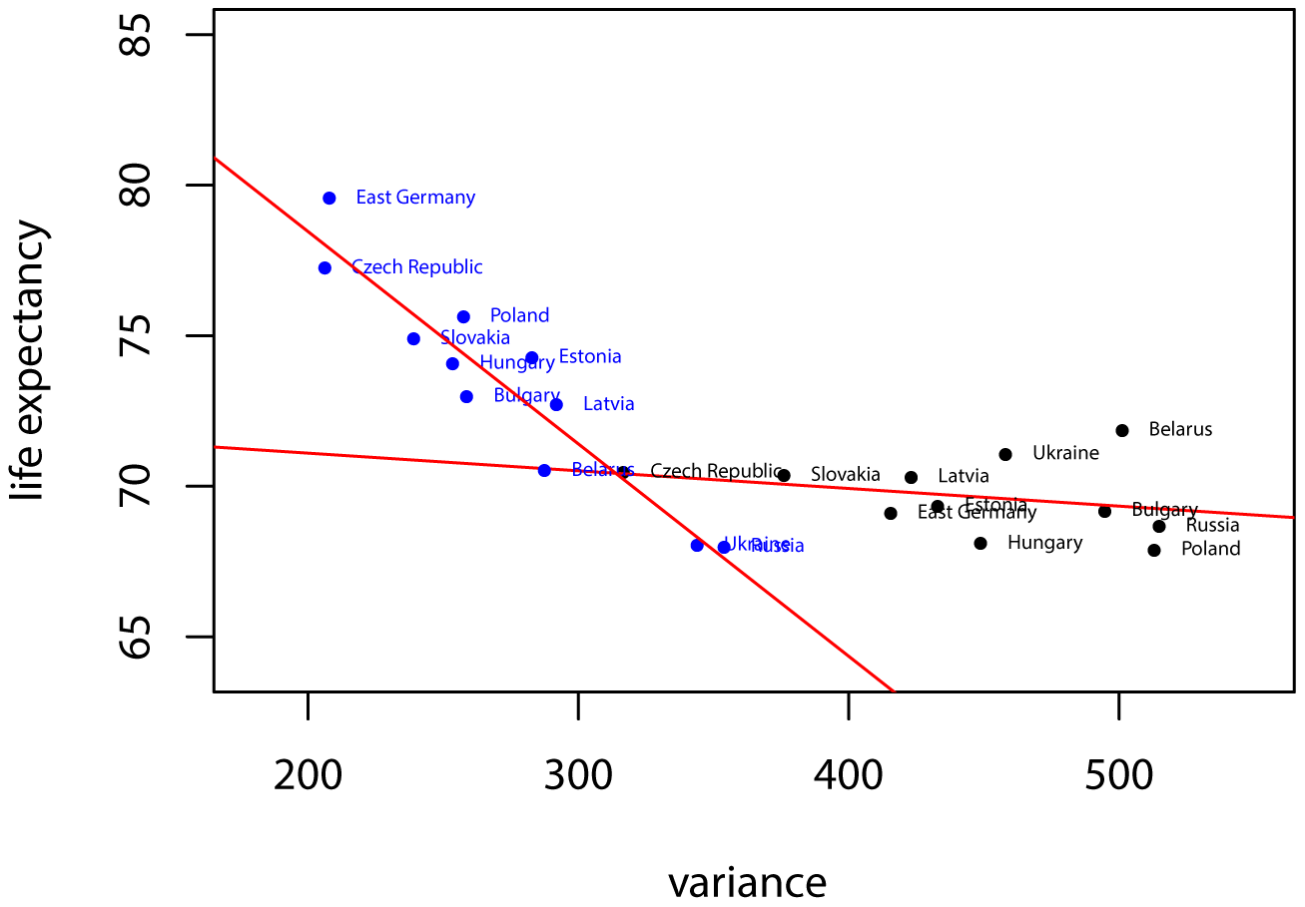
Variance and life expectancy profiles of Eastern countries. In blue, the variance and life expectancy profile of Eastern countries in the year 2008 and in black for the year 1960.

Our first set of results, before we turn to the KLD trends, are based on the traditional mean and variance study of the mortality distribution. We provide a graphical analysis of the mean and variance of the ages-at-death distributions over time that also uncovers some interesting features of the epidemological transition. Figures 1 and 2 plot the samples of Western and Eastern countries in the mean-variance space for the years 1960 and 2008.

A remarkable feature of the data is the organization of Western countries in 1960 is mostly along a line that orders countries in the space of high life expectancy — low inequality and low life expectancy — high inequality. This correlation reflects the reality of countries in different stages of their epidemiological transitions. After 50 years, the picture that emerges is drastically different. Although there have been common trends among the majority of countries, i.e. the generalized reduction of variance and the increase in life expectancy, the resulting distribution of countries encompasses a heterogenous landscape. The previous correlation is less apparent, with countries with new profiles emerging from the distribution. For instance, we observe countries with similar variances (Denmark, Australia, Japan) but with large differences in life expectancy. Performing a similar exercise for Eastern countries yields an interesting contrast. In the latter case, the historical evolution is reversed, with countries evolving from many different profiles to the single dimension ordering as seen amongst Western countries in the 1960's. The mean-variance profiles we report are coherent with previous work [13], [14] that relates the relationship between life expectancy and variance to the different stages of the epidemiological transition.

In the next section we provide an evaluation of convergence based on the KLD. The main strength of the KLD is that it encompasses the whole differences in distributions, providing a clearer picture than analysis based on only a collection of moments. This is particularly relevant for the analysis of the mortality distributions as infant mortality breaks the normality of the ages-at-death distribution. The ages-at-death distributions of the earlier periods contained a considerably larger number of infant deaths than the contemporary distributions, making mean-variance comparisons a less accurate evaluation.

### KLD trends

**Figure 3:**
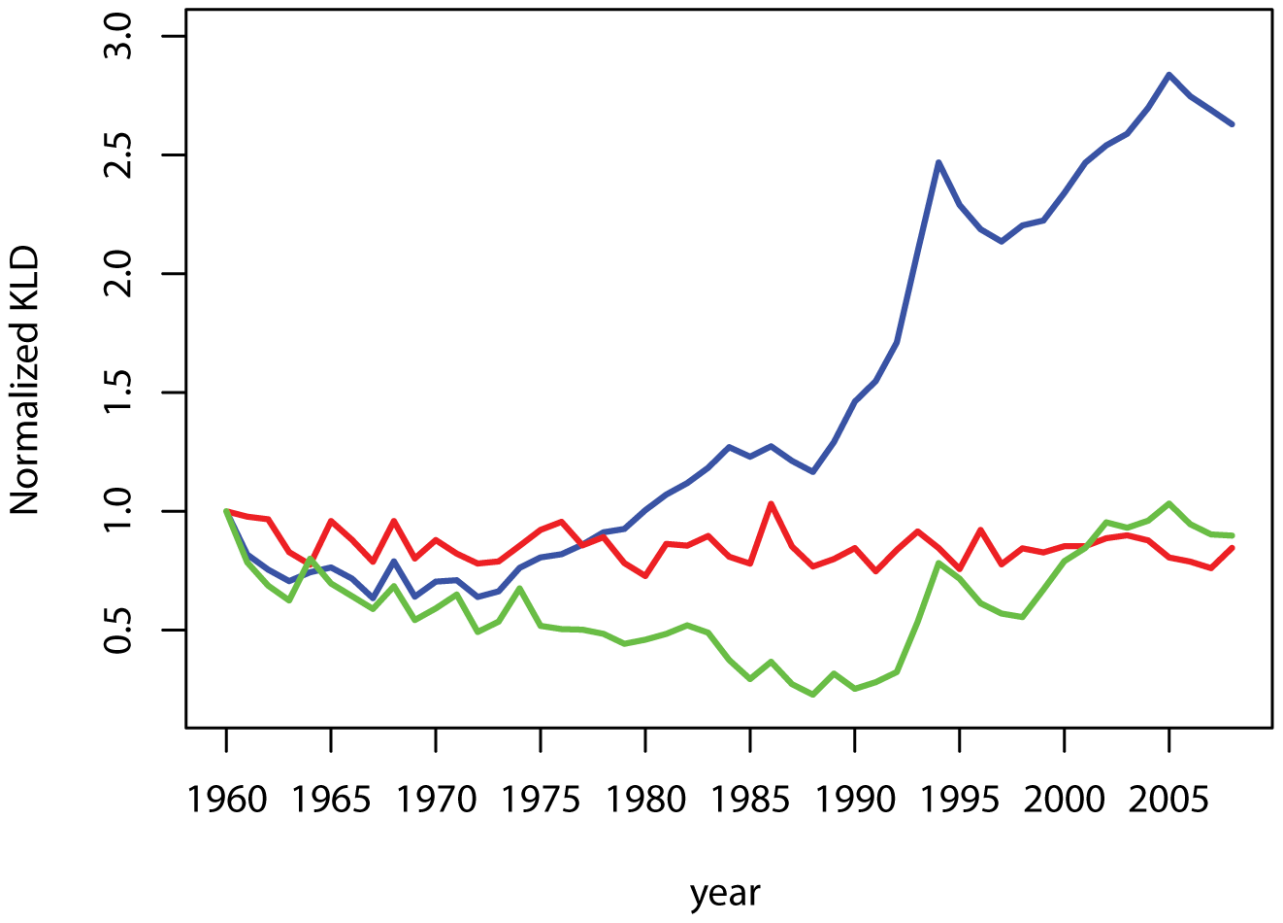
Disparities across mortality distributions. The sum of KLD divergences to the mean ages-at-death distribution is normalized with reference to the starting value in 1960. The KLD for the whole sample is in blue, the Eastern countries in green and Western countries are in red.

**Figure 4:**
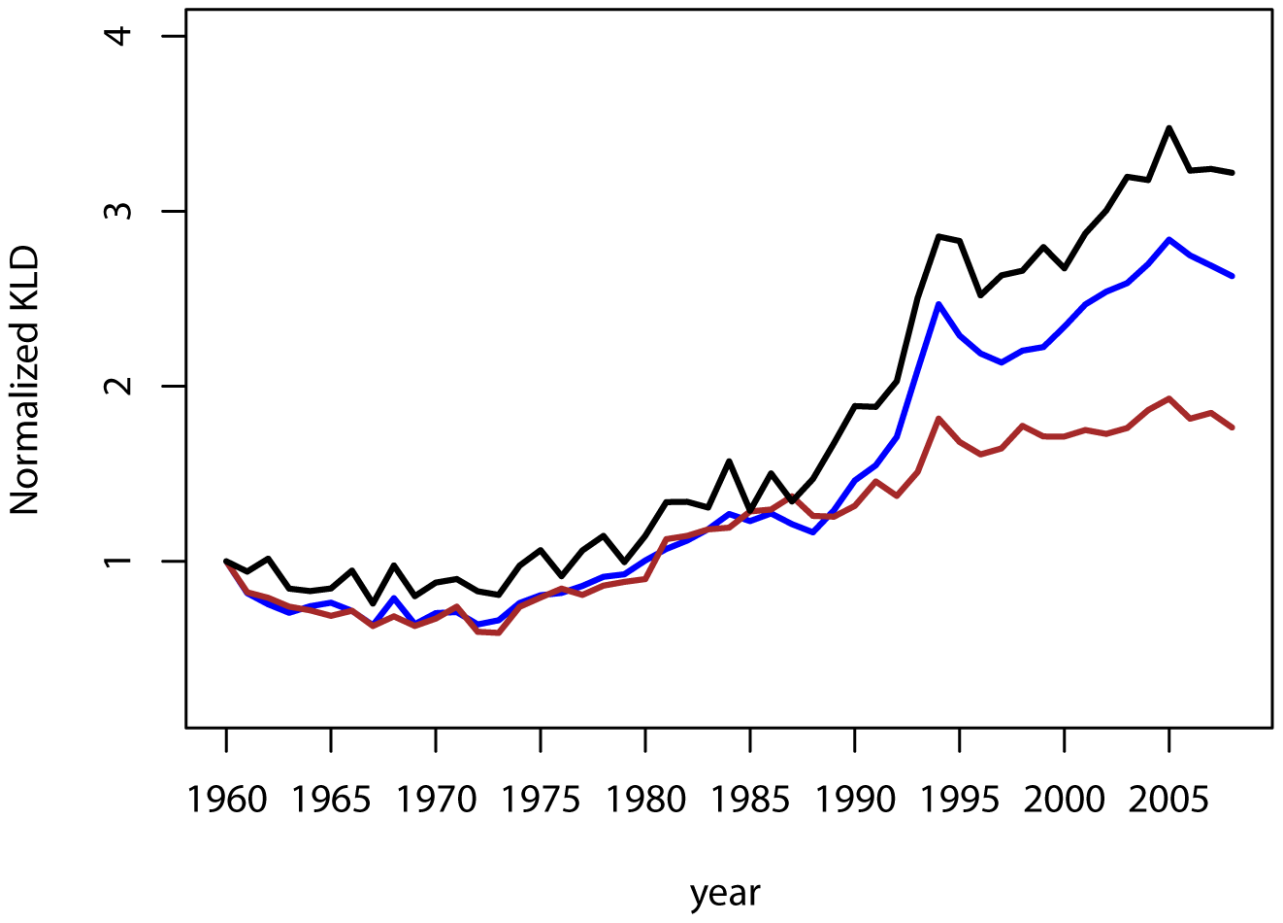
Disparities across mortality distributions by gender. The sum of KLD divergences to the mean ages-at-death distribution is normalized with reference to the starting value in 1960. The KLD for the whole sample is in blue, the female-only in brown and male-only in black.

Our results indicate a clear pattern of divergence in our sample of industrialized countries (Figure 3). The KLD diminishes from the 1960's until the 1970's, then rises over the 1980's. In fact, the KLD more than doubles by the end of the 1980's, a period that coincides with the dissolution of the Soviet Union, before returning to the positive trend similar to the earlier part of the 1980's. The trends reported remain very similar for both genders, lending support to the robustness of our findings (Figure 4). It is of note that the increase in male disparities is of a larger magnitude than that of the female distribution. While by 2008 the female's sample KLD is 1.76 larger that in 1960, the male's sample KLD is over 3 times larger.

The relevance of taking into account the existence of clubs of countries becomes apparent when observing the differences in the trends between the two groups of countries. In the sample of Western countries we find a relatively flat profile of convergence for mortality patterns (Figure 3); that is, no reduction in the overall differences in mortality patterns. Our conclusion of no convergence is mainly based on the comparison with the strength of the other trends we report. Given the volatility of the KLD a statistical analysis of the trend is very sensitive to the period considered. The sample of Eastern countries (Figure 3) reveals, as expected, a break immediately following the dissolution of the Soviet Union. The first 30 years are marked by convergence, whereas in the late 1980's the trend is completely reversed and we observe a large increase in the differences, followed by sustained increases in the KLD.

## Discussion

Our main finding suggests that, while the reduction in differences due to the catching up process of countries at earlier stages of development is an important catalyst towards convergence [2], in the later stages structural differences between countries have emerged and halted the convergence process, with young adult mortality differences playing an important role [9]. Based on previous research that provided evidence of a mortality shock in former Soviet countries, we investigate the trajectories of two distinct groups of countries, Eastern and Western countries. Our observations on Eastern countries, in line with previous studies [15], point out that structural sources of differences may be strong enough to set back the realized gains in life expectancy. We also find evidence of the lack of a robust convergence process even amongst Western countries.

While our results uncover group specific trends, our classification of countries does not identify convergence clubs. There exists a period of convergence for Eastern countries prior to the 1990s but the recent history of development shows that Eastern countries are no longer approaching a common distribution. Given the robustness of the lack of overall convergence and the crucial role that group-specific dynamics play, further research is needed to uncover the determinants of club membership and pertinence.

The discussion on the determinants of club membership can be divided into two areas of study depending on whether the focus is on the differences between developed and developing countries or the differences amongst industrialized countries. When focusing on the former, the literature has highlighted the existence of mortality traps [7]. That is, there is evidence that there might exist two types or clubs of countries, ones with low life expectancy and low growth of life expectancy and ones with high life expectancy and high growth of life expectancy. In order to transition from one club to another, a certain threshold of life expectancy must be reached. However, the variables that define a club still remain relatively unexplored. A first step in the search for these structural conditions can be to draw from the rich literature on the historical path of mortality transitions for current high life expectancy countries [16]. When considering industrialized countries, likely suspects are the variables that influence young adult mortality. Given that a large part of the disparities across countries in mortality patterns can be attributed to young adult mortality [9], it is reasonable to believe that the variables influencing mortality for those age groups might play an important role in determining the formation of convergence clubs.
